# A Manual of the Diseases of the Eye

**Published:** 1846-03

**Authors:** 


*A Manual of the Diseases of the Eye.* By S. Littell, M. D. One of the Surgeons of the Wills' Hospital; Fellow of the College of Physicians of Philadelphia, &c. &c. Second edition. Revised and Enlarged.

The first edition of Dr. Littell's Manual was published in 1837, and contained 250 pages. Its favorable reception was attested by its republication in London, under the auspices of Dr. Houston, a distinction not often awarded to American works, notwithstanding our avidity in seizing upon the books of our transatlantic brethren, and dressing them up to suit the home market—a practice, by the by, rather derogatory to native talent, and calculated, we fear, to retard the advancement of the medical literature of our own country. The present volume is got up in a style quite superior to its predecessor, besides being enlarged to the dimensions of 372 pages.

The chief merit of the work of Dr. Littell is its conciseness and perspicuity—embracing, as it does, a description of all the important diseases of the eye and its appendages—without the multiplicity of names, the useless distinctions, and the elaborate and verbose disquisitions which distinguish the productions of some foreign writers on Ophthalmology.

The author's connection with the Wills' Hospital for the lame and blind, has given him opportunities for observation and study in this department of surgery which few can enjoy; and we are happy to find that he has frequently introduced into his Manual the results of the experience of himself and colleagues upon important questions of ophthalmic practice. In treating of the operation for cataract, we find the following instructive remarks, which will give the reader an idea of the author's style, and of the practical tendency of his work.

"The operation is performed in three different modes,—extraction, depression, and division or absorption,—each of which possesses advantages which may render it eligible under certain circumstances.

In this country the operation by *absorption* is generally preferred; it is well adapted to the removal of soft or fluid cataract, the usual form in which the disease appears in early or middle life; requires little manual dexterity; is attended with less danger to the eye than either of the others; and may be employed in a multitude of cases where, from complications and other causes, extraction is inapplicable. The principle on which it is founded, is the removal of the lens by the agency of the aqueous humour, admitted through an opening in the capsule. The pupil having been previously dilated by the application of belladonna to the brow, and the patient placed upon a table in a supine position, supported by pillows, an assistant retracts one of the lids, while the surgeon takes charge of the other, and introduces a sharp-edged needle,—either straight or very slightly curved, and about eight or ten lines long in its shaft,—through the sclerotica, parallel with its fibres, a little above its transverse diameter, and not more than a line from the cornea. The point of the instrument having been directed towards the centre of the eye while piercing the sclerotica, should be brought forward as soon as it has fairly penetrated that membrane, by depressing the handle towards the temple, and after being rotated one-fourth of a revolution, so as to bring its flat surface upwards, carried between the fringed extremity of the ciliary processes and the circumference of the capsule, across the posterior chamber, a little beyond the middle of the pupil. If the needle have penetrated the eye too deeply in the first instance, it should be partially withdrawn, else it would be entangled in the substance of the lens; and, while endeavouring to avoid this accident on the one hand, some care is necessary not to injure the iris on the other. When the instrument, thus dexterously guided through the narrow passage, has fairly reached the centre of the pupil, its cutting edge is turned towards the opacity, and the capsule with the crystalline gently divided into several pieces. In performing this part of the operation, it is advisable to raise the needle repeatedly clear of the cataract, and re-enter it again in a different part, but not to raise the point too near its superior margin, otherwise it would be liable to be dislocated, and revolve upon its axis;—a circumstance, however, from which no great mischief would arise, except, perhaps, a greater tendency to inflammation from the pressure upon the iris. If the lens happen to be fluid, it escapes immediately on opening the capsule, and is diffused through the aqueous humour; and when it is of firmer consistence, small fragments are often observed floating through the chambers.

In those instances where the cataract is soft or fluid, a single operation is sufficient for its removal; but under other circumstances it is often necessary to repeat it after an interval of six or eight weeks. On these subsequent occasions, the crystalline should be more completely comminuted, and the fragments passed into the anterior chamber, where absorption is supposed to take place more rapidly;—care being taken to avoid, if possible, the posterior hemisphere of the capsule.

The mode of operation just described, is that usually adopted at the Wills Hospital, both by the author and his colleagues, and has been followed by a remarkable degree of success; the consequence, probably, of the great attention which is given, both in the preparatory and subsequent treatment, to the prevention of inflammation."

There is another point in which the work of Dr. Littell differs widely from some of the late publications on ophthalmology, and which, in our judgment, forms one of its strongest recommendations. Undue prominence is not given to the description of operative procedures upon the eye, to the neglect of the more ordinary diseases of the organ which are constantly occurring to the practical physician, and about which he desires to be informed. Long descriptions of the various methods of operating adopted by different surgeons, with plates of instruments, &c., many of which are never brought into use, may serve to amuse the eye, and swell out the pages of a book, but they can have no practical benefit. The late popular operation of strabismus, for instance, which has occupied so much space in some of the recent works on diseases of the eye, is disposed of by our author in four and a half pages; and we must be allowed to say that, to our mind, he has in this short space presented a more philosophical view of the nature of this deformity, and of its means of cure, than is contained in some of the long treatises to which we have referred. It is true that our author does not undertake to enter into detail upon this or any other subject, in what professes to be a "Manual," while, at the same time, he has generalized so judiciously, that his "little volume" will, we think, be found quite as instructive, both to practitioners and students, as many of the more voluminous works on the same subjects.

We have long believed that one cause of the ignorance which prevails in the great mass of the profession, upon this department of surgery, arises from the forbidding aspect of the books which treat specially of it. It is looked upon as a sort of insurmountable speciality, which requires long and arduous study, to master all its details. Its terminology alone excites alarm, and warns the student of its approach, and hence he too often abandons it in despair. If the work of Dr. Littell should tend to divest the subject of these difficulties, and to present it in a more simple and attractive form, he will have performed a service to the profession by no means unimportant.

It is, therefore, with great pleasure, that we recommend the volume before us, both to the practical physician and the student; and although it may not take the place of the larger works of Lawrence, Mackenzie, Middlemore, Morgan, and others, it is yet an admirable treatise, which does great credit to the author, and cannot but prove an acceptable offering to the profession.

